# Expression of the costimulatory molecule B7-H3 is associated with prolonged survival in human pancreatic cancer

**DOI:** 10.1186/1471-2407-9-463

**Published:** 2009-12-26

**Authors:** Martin Loos, Dennis M Hedderich, Malte Ottenhausen, Nathalia A Giese, Melanie Laschinger, Irene Esposito, Jörg Kleeff, Helmut Friess

**Affiliations:** 1 Department of Surgery, Klinikum rechts der Isar, Technische Universität München, Ismaninger Str 22, 81675 Munich, Germany; 2 Department of Pathology, Klinikum rechts der Isar, Technische Universität München, Ismaninger Str 22, 81675 Munich, Germany; 3 Department of General Surgery, University of Heidelberg, Im Neuenheimer Feld 110, 69120, Heidelberg, Germany

## Abstract

**Background:**

Costimulatory signaling has been implicated as a potential regulator of antitumor immunity in various human cancers. In contrast to the negative prognostic value of aberrant B7-H1 expression by pancreatic cancer cells, the role of B7-H3 is still unknown. Therefore, we investigated the expression pattern and clinical significance of B7-H3 expression in human pancreatic cancer.

**Methods:**

B7-H3 expression was evaluated by immunohistochemistry in 68 patients with pancreatic cancer who underwent surgical tumor resection. Expression data was correlated with clinicopathologic features and with the number of tumor-infiltrating T cells.

**Results:**

B7-H3 expression was significantly upregulated in pancreatic cancer compared to normal pancreas (p < 0.05). In 60 of 68 examined tumors B7-H3 protein was detectable in pancreatic cancer cells. Patients with high tumor B7-H3 levels had a significantly better postoperative prognosis than patients with low tumor B7-H3 levels (p = 0.0067). Furthermore, tumor B7-H3 expression significantly correlated with the number of tumor-infiltrating CD8+ T cells (p = 0.018).

**Conclusion:**

We demonstrate for the first time that B7-H3 is abundantly expressed in pancreatic cancer and that tumor-associated B7-H3 expression significantly correlates with prolonged postoperative survival. Our findings suggest that B7-H3 might play an important role as a potential stimulator of antitumor immune response in pancreatic cancer.

## Background

Pancreatic cancer is one of the most devastating human malignancies. At present, pancreatic cancer is the fourth leading cause of cancer-related deaths in the U.S. [1]. Despite great efforts on clinical and molecular pancreatic cancer research, the prognosis remains poor with an overall 5-year-survival of less than 5% [2]. Complete surgical resection still represents the only potentially curative treatment [3]. By the time of diagnosis, however, only few patients are candidates for surgery.

The capability to modulate or even suppress antitumor immune responses is a characteristic feature enabling malignant tumors to evade immune surveillance and subsequent destruction [4-6]. Costimulatory signaling plays a key role in the initiation and termination of immune responses by regulation of T cell priming, growth, maturation, and tolerance [7]. One of the best characterized costimulatory pathways includes B7-1/B7-2:CD28 signaling [7]. Engagement of B7-1 on antigen-presenting cells with CD28 on T cells enhances T cell proliferation and IL-2 production. In the absence of this simultaneous costimulatory signal, ligation of the T cell receptor by an antigenic peptide results in T cell dysfunction, intolerance or anergy [7]. Beside positive costimulatory signals that augment and sustain T cell response, costimulatory pathways also deliver inhibitory signals which downregulate T cell response. Binding of CTLA-4 to B7-1 and/or B7-2 has been shown to inhibit IL-2 synthesis and progression through the cell cycle leading to the termination of T cell response [7,8].

Within the past decade, new costimulatory ligands and receptors have been identified including B7-H1 (programmed death-1 ligand-1), B7-DC (programmed death-1 ligand-2), PD-1 (programmed death-1), B7-H3, and B7-H4 [9,10]. In contrast to the well-defined roles of B7-1 and B7-2 as essential costimulators involved in T cell activation, the function of B7-H1, B7-DC, B7-H3, and B7-H4 is less clear. Recently, B7 homologues have been implicated as potential regulators of antitumor immunity [4]. Numerous human cancers have been reported to aberrantly express B7-H1 [11-14]. Aberrant B7-H1 expression by tumor cells has been associated with adverse pathologic features and poor outcome [15,16]. We and others have previously shown that pancreatic cancer patients with cancer-cell associated B7-H1 expression had a significantly poorer prognosis than patients with B7-H1 negative tumors [12,13]. Thus, aberrant expression of B7-H1 has been postulated as a potential mechanism by which malignant tumors may evade the host immune response. A possible explanation for the tumor-associated immune suppression is that aberrant expression of B7-H1 might downregulate tumor-specific T cell response by inducing T cell anergy or apoptosis.

In contrast to the negative prognostic value of aberrant B7-H1 expression by tumor cells, the role of B7-H3 in human cancers is controversial. B7-H3 is a B7 homologue that shares approximately 25% sequence homology with B7-H1. In contrast to B7-H3 mRNA, which is broadly expressed in lymphoid and non-lymphoid organs, B7-H3 protein expression seems to be limited. Functionally, B7-H3 is thought to serve as an accessory costimulatory regulator of T cell responses following initial T cell priming. However, its exact physiologic role is still unclear, because both stimulatory and inhibitory properties have been described. Initially, it has been reported that human B7-H3 costimulates proliferation of both CD4+ and CD8+ T cells, enhances the induction of cytotoxic T cells, and selectively stimulates IFN-γ production in the presence of T cell receptor signaling [17]. In mice, however, B7-H3 inhibits proliferation of both CD4+ and CD8+ T cells and IFN-γ production [18]. In accordance to its inconsistent immunologic function, the role of B7-H3 in cancer remains far from clear. For example, transfection of B7-H3 into P815 tumor cells led to the regression of tumors and amplification of a tumor-specific CD8+ cytotoxic lymphocyte response in syngeneic mice [19]. In another experimental study, adenoviral B7-H3 treatment resulted in a reduction of tumor size and a significant reduction of secondary metastases in an orthotopic murine colon cancer model [20]. In a model of EL-4 lymphoma, injection of a B7-H3 expression plasmid into the tumor resulted in complete regression of 50% of tumors [21]. The response depended upon CD8+ T cells and NK cells [21]. In contrast to these tumor-protective effects of B7-H3, tumor B7-H3 expression in human non-small-cell lung cancer inversely correlated with the number of tumor-infiltrating lymphocytes and significantly correlated with lymph node metastases [22]. In prostate cancer, two studies analyzing B7-H3 expression in a considerable number of patients revealed that almost all examined tumors expressed B7-H3 protein. Both studies showed a significant correlation between high B7-H3 expression by tumor cells and disease spread and poor outcome [23,24].

Here, we show for the first time that B7-H3 is abundantly expressed in pancreatic cancer and that B7-H3 expression by pancreatic cancer cells correlates with increased patient survival as well as with the number of tumor-infiltrating CD8+ T cells.

## Methods

### Patients

All tissue specimens analyzed in this study were obtained according to institutional review board-approved procedures for consent. Human pancreatic cancer tissue samples were obtained from 96 patients (39 female and 57 male patients with a median age of 63 years; age range, 38-83 years), who underwent resection for pancreatic cancer at the University Hospital of Heidelberg (Heidelberg, Germany) between 2001 and 2006. Two patients presented with stage IB, 11 patients presented with stage IIA disease, 70 patients presented with stage IIB disease, two patients presented with stage III disease, and 11 patients presented with stage IV disease according to the UICC classification (6th edition, 2002). The median follow-up was 23 months, with a range from 2 to 44 months. Normal human pancreatic tissue samples were obtained through an organ donor program from 10 previously healthy individuals (four female, six male; median age of 43 years, with a range of 17-62 years) when there was no suitable recipient.

### Tissue Sampling

Immediately after surgical removal, tissue samples were either snap-frozen in liquid nitrogen (for RNA and protein extraction) or fixed in 10% buffered formalin solution and embedded in paraffin for histological analysis. A serial section of each specimen was stained with H&E for histological evaluation. In addition, a portion of the tissue specimen was preserved in RNAlater (Ambion Europe Ltd., Huntington, Ambridgeshire, UK).

### Cell culture

Panc-1, MiaPaCa-2 and SU86.86 pancreatic cancer cells were grown routinely in RPMI 1640 with L-glutamine and 25 mM HEPES (Gibco, Karlsruhe, Germany) supplemented with 10% fetal bovine serum (FBS), 100 U/ml penicillin, and 100 μg/ml streptomycin (Invitrogen, Karlsruhe, Germany) and incubated in a 5% CO_2 _humidified atmosphere.

### Quantitative RT-PCR

All reagents and equipment for mRNA/cDNA preparation were purchased from Roche Applied Science (Mannheim, Germany). Messenger-RNA was prepared by automated isolation using MagNA Pure LC instrument and isolation kits I (for cells) and II (for tissues). Complementary-DNA was prepared using the First Strand cDNA Synthesis Kit for reverse transcription-PCR according to the manufacturer's instructions. Real-time PCR was performed with the LightCyclerFastStart DNA SYBR Green kit as described previously [25]. B7-H3, CD4, CD8, and IFN-γ primers were obtained from Search-LC (Heidelberg, Germany). The number of specific transcripts was normalized to the average expression of two housekeeping genes (cyclophillin B and hypoxanthine-guanine phosphoribosyltransferase) [25]. The data of two independent analyses for each sample and parameter were averaged and presented as adjusted transcripts/μl cDNA.

### Antibodies/Immunohistochemistry

Immunohistochemical analyses were carried out with mouse anti-human B7-H3 antibody (MAB 1027, R&D Systems, Minneapolis, MN), mouse anti-human CD4 antibody (1F6, Monosan, Uden, Netherlands), and mouse anti-human CD8 antibody (DAKO Diagnostics AG, Zürich, Switzerland). Antigen retrieval was achieved by microwave pre-treatment in citrate buffer. Paraffin-embedded pancreatic tissue sections (3-μm thick) were deparaffinized with xylene and rehydrated through graded alcohol into distilled water. After washing in Tris-buffered saline, endogenous peroxidase activity was quenched by incubating the slides in 3% hydrogen peroxide in methanol. To block unspecific activity of secondary antibodies, slides were treated with TBS/3%BSA. After overnight incubation at 4°C with the primary antibody, slides were washed with Tris-buffered saline and 0.05% Tween 20, treated with anti-mouse (DAKO) horseradish peroxidase-labeled secondary antibody for 1 hour, developed using Dako Envision System (DAKO), and counterstained with Mayer's hematoxylin. To ensure antibody specificity, negative control slides were incubated with normal mouse IgG. Immunohistochemistry results were evaluated by scanning each slide under low power magnification (×10) to identify regions containing positive immunoreactivity. Immunostaining was further evaluated at high power magnification (×200). Tumor samples were examined by two observers in a blind manner.

### Semi-quantitative analysis of B7-H3 expression in tissues and its correlation with patient survival

To assess the impact of B7-H3 protein expression by cancer cells on patient survival, tissue blocks of 68 histologically confirmed pancreatic cancer patients were analyzed by immunohistochemistry. Evaluation of B7-H3 staining in cancer cells was made semi-quantitatively as published by Erkan M and co-workers [25]. Scores were given separately for the stained area and for the intensity of staining. Quantification was made as follows; ≤33% of the cancer cells: 1, >33 to ≤66% of the cancer cells: 2, >66% of the cancer cells: 3; intensity of staining: absent/weak: 1, moderate: 2, strong: 3. Each section had a final grade that derived from the multiplication of the area and intensity scores. Sections with a final score of ≤3 were classified as tumors with low B7-H3 expression, whereas sections with a final score of > 3 were classified as tumors with high B7-H3 expression.

### Semi-quantitative evaluation of the prevalence of tumor-infiltrating CD4+ and CD8+ T cells in pancreatic cancer tissues and correlation with B7-H3 expression

To examine whether tumor B7-H3 expression is associated with the infiltration of CD4+ or CD8+ T cells, we semi-quantitatively evaluated the prevalence of CD4+ and CD8+ cells in pancreatic cancer specimens. Consecutive slides from the same tumor were stained for CD4, CD8, and B7-H3. Using histological landmarks, corresponding areas with tumor B7-H3 and without tumor B7-H3 expression were identified under low power magnification (×10). The prevalence of CD4+ or CD8+ T cells was semi-quantitatively evaluated at high power magnification (×400) according to the number of CD4 positive or CD8 positive cells per areas: Areas with 0-5 CD4 positive or CD8 positive cells were recorded as (0), areas with 6-15 CD4 positive or CD8 positive cells were recorded as (1), and areas with >15 CD4 positive or CD8 positive cells were recorded as (2). Tumor samples were examined by two observers in a blind manner.

### Induction of B7-H3 in cultured pancreatic cancer cell lines and FACS analysis

To determine the effect of IFN-γ (R&D Systems) or IL-4 (R&D Systems) on the expression of B7-H3 in cultured pancreatic cancer cells, Panc-1, MiaPaCa-2, and SU86.86 cells were seeded in 6-well plates (100,000 cells per well), and incubated with 2000 IU/ml of recombinant IFN-γ or 40 ng/ml of recombinant IL-4 for 24 or 48 hours. Cells were harvested and stained with anti-human B7-H3-FITC mAb (R&D Systems) or control anti-human IgG-FITC (BD Pharmingen, Heidelberg, Germany). Single-color flow cytometry was performed by using a FACSCalibur (Becton-Dickinson, BD) and analyzed with FlowJo Software (Tree Star).

### Statistical Analysis

Results are expressed as mean ± SD (mRNA expression). For statistical analysis, GraphPad Prism software (GraphPad Software, San Diego, CA) was used. The Mann-Whitney U test, χ^2 ^test or Spearman rho test were performed for comparative statistical evaluations among groups and for correlation analysis with histological and clinical parameters (age, gender, tumor stage, tumor grade, and postoperative survival). Significance was defined as p < 0.05. The Kaplan-Meier method was used to estimate the probability of survival, and significance was assessed by the log-rank test.

## Results

### Expression of the costimulatory molecule B7-H3 in human pancreatic cancer

**Figure 1:**
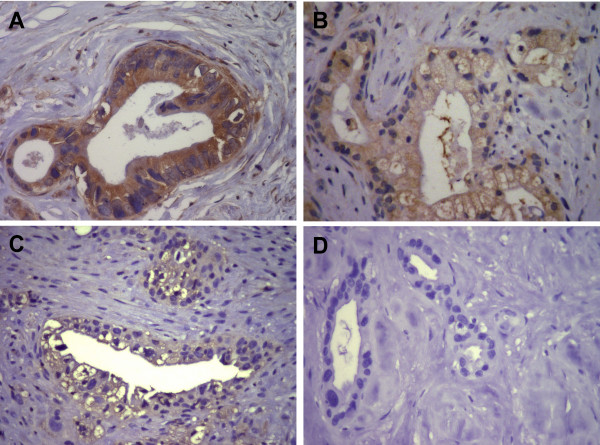
**Immunohistochemical characterization of B7-H3 expression in 4 representative human pancreatic cancer tissue sections**. Positive B7-H3 immunoreactivity was detectable in pancreatic cancer cells (brown staining). Representative tissue sections of strong intensity (A), moderate intensity (B), and weak intensity (C). (D) Negative control.

Quantitative RT-PCR was performed to assess mRNA expression of the costimulatory molecule B7-H3 in 28 surgically resected human pancreatic cancer tissue specimens and 10 normal human pancreatic tissue specimens. B7-H3 mRNA was significantly upregulated in pancreatic cancer tissues (275.5 transcripts ± 44.4) compared to normal pancreatic tissue samples (86.5 ± 22.2; p < 0.05). To confirm the qRT-PCR data and to assess the location of B7-H3 protein, we performed immunostainings of 68 pancreatic cancer tissue samples. In pancreatic cancer tissues, abundant positive B7-H3 immunoreactivity could be detected in pancreatic cancer cells (Fig. 1), pancreatic islet cells, tumor-infiltrating immune cells, and sporadical weak immunoreactivity in tumor-surrounding tubular complexes and endothelial cells. Among the examined 68 pancreatic cancer tissues, 60 showed positive B7-H3 protein expression in pancreatic cancer cells. According to the staining intensity, all specimens were classified into three groups with either no or weak, moderate or strong immunoreactivity (Fig. 1). Depending on the area of positive immunoreactivity, a final overall score (high tumor B7-H3 or low tumor B7-H3 expression) was established as described in the methods section. In normal pancreatic tissues, weak B7-H3 protein expression could be sporadically detected in normal pancreatic acinar and ductal cells.

### B7-H3 expression correlates with better postoperative survival in pancreatic cancer patients

**Figure 2:**
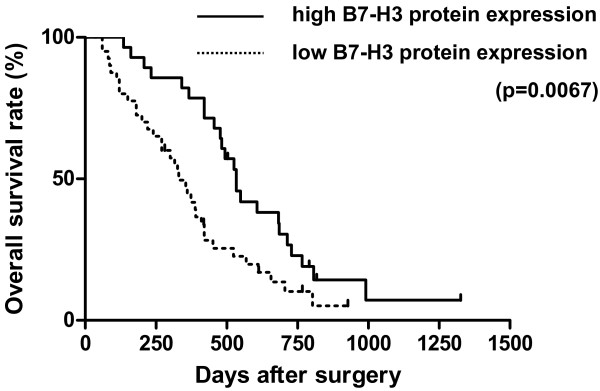
**Overall survival of 68 pancreatic cancer patients in relation with B7-H3 protein expression**. Patients with high tumor B7-H3 protein expression had a significantly better prognosis than patients without or with low tumor B7-H3 protein expression (p = 0.0067). The median survival of patients with high tumor B7-H3 protein expression was 17.8 months in contrast to 11.0 months in patients without or with low tumor B7-H3 protein expression.

Costimulatory signaling has been implicated as a possible regulator of antitumor immunity in several human malignancies. We investigated the relationship between tumor-associated B7-H3 protein expression and various clinicopathological features in pancreatic cancer (Table 1). No correlation could be observed between tumor B7-H3 expression and age, gender, tumor stage, and tumor grade. In contrast, high tumor B7-H3 expression was associated with significantly prolonged postoperative survival in pancreatic cancer patients (p = 0.0067; Fig. 2). Among the examined 68 pancreatic cancer tissues, 40 showed low tumor B7-H3 expression, whereas 28 showed high tumor B7-H3 expression. The median survival in patients with high tumor B7-H3 expression was 17.8 months and 11.0 months in patients with low B7-H3 expression (Fig. 2).

**Table 1:** Clinicopathological characteristics according to tumor B7-H3 expression.

		**B7-H3 expression**		
**Variable**	**n**	**low**	**high**	**p-value**
Gender				n.s.
Female	24	16	8	
Male	44	25	19	
				
Tumor status				n.s.
T1	0	0	0	
T2	4	3	1	
T3	62	36	26	
T4	2	1	1	
				
Nodal status				n.s.
N0	10	5	5	
N1	58	35	23	
				
Metastatic status				n.s.
M0	58	35	23	
M1	10	4	6	
				
Tumor grade				n.s.
G1	2	1	1	
G2	44	27	17	
G3	22	12	10	
G4	0	0	0	
				
Survival (in months)		11	17,8	p = 0.0067

### IFN-γ is upregulated in human pancreatic cancer in vivo and significantly correlates with tumor B7-H3 expression

**Figure 3:**
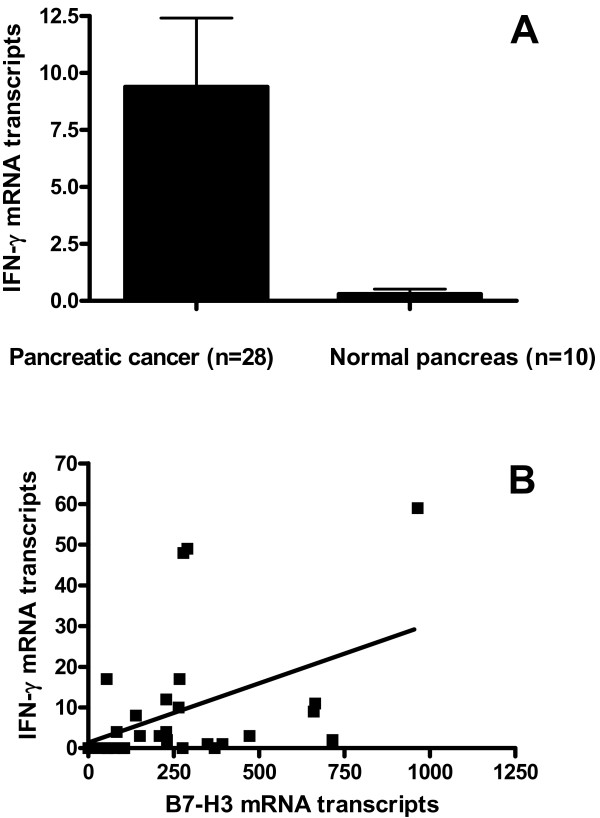
**IFN-γ mRNA expression in 28 pancreatic cancer tissues and 10 normal pancreatic tissues**. (A) IFN-γ mRNA expression was significantly upregulated in pancreatic cancer tissues compared to normal pancreas (p < 0.05). (B) Correlation analysis of B7-H3 mRNA and IFN-γ mRNA expression in pancreatic cancer (n = 28) revealed a positive correlation between B7-H3 and IFN-γ mRNA expression (p = 0.0225; Spearman rho 0.4297).

In vitro, B7-H3 has been shown to selectively stimulate IFN-γ production by purified human T lymphocytes [17]. To examine whether B7-H3 expression is associated with IFN-γ levels in vivo, we analyzed cytokine gene expression of B7-H3 and IFN-γ by qRT-PCR in 28 pancreatic cancer tissue samples and 10 normal pancreatic tissue samples. Gene expression of IFN-γ was significantly upregulated in pancreatic cancer tissues in comparison to normal pancreatic tissues (p < 0.05; Fig. 3A). Correlation analysis revealed a statistically significant correlation between B7-H3 and IFN-γ expression (p = 0.0225; Spearman rho 0.4297; Fig. 3B).

### Cultured pancreatic cancer cell lines Panc-1, MiaPaCa-2, and SU86.86 constitutively express B7-H3

To test whether pancreatic cancer cells express the costimulatory molecule B7-H3 in vitro, we performed qRT-PCR. All tested cultured pancreatic cancer cell lines constitutively expressed B7-H3 mRNA under normal conditions (Panc-1 541 transcripts ± 36.6, MiaPaCa-2 358 transcripts ± 17.5, SU86.86 743 transcripts ± 725).

### Treatment of cultured SU86.86 pancreatic cancer cells with IFN-γ or IL-4 markedly increases their B7-H3 expression

**Figure 4:**
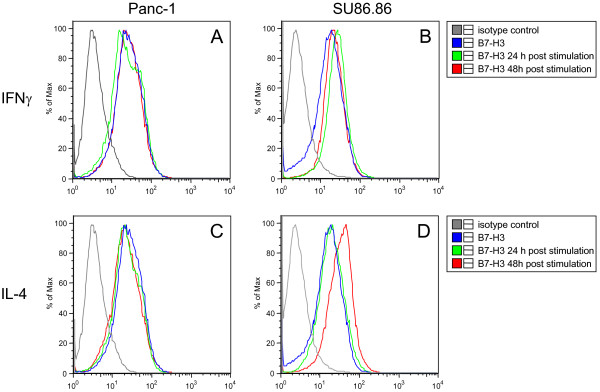
**Effects of treatment with IFN-γ or IL-4 on Panc-1 and SU86.86 pancreatic cancer cells**. Cells were treated with either 2000 IU/ml of recombinant IFN-γ or 40 ng/ml of recombinant IL-4 for 24 or 48 hours and analyzed by FACS. (B) IFN-γ treatment resulted in a marked increase of B7-H3 expression on SU86.86 cells after 24 hours (green line) and 48 hours (red line). (D) IL-4 treatment resulted in a marked increase of B7-H3 expression on SU86.86 cells after 48 hours (red line), but not after 24 hours (green line). In contrast, (A) IFN-γ treatment led to a slight decrease of B7-H3 expression on Panc-1 cells 24 hours after stimulation (green line) with normalization 48 hours after stimulation (red line). (C) IL-4 treatment led to a slight decrease of B7-H3 expression on Panc-1 cells 24 hours after stimulation (green line) and 48 hours after stimulation (red line). The blue lines display B7-H3 expression without stimulation by IFN-γ and IL-4. The grey lines display the (isotope) control group.

To assess the effect of the Th1 and Th2 cytokines IFN-γ and IL-4 on B7-H3 expression by cultured pancreatic cancer cells, cells (representative pancreatic cancer cell lines Panc-1 and SU86.86) were incubated with recombinant IFN-γ and IL-4 for 24 and 48 hours. Treatment of Panc-1 cells with IFN-γ slightly decreased the expression of B7-H3 after 24 hours. The effect nearly disappeared after 48 hours (Fig. 4A). In SU86.86 cells IFN-γ treatment resulted in a marked increase of B7-H3 expression after 24 hours which slightly decreased after 48 hours (Fig. 4B). IL-4 treatment similarly led to a slight decrease of B7-H3 expression in Panc-1 cells after 24 hours which was more pronounced after 48 hours (Fig. 4C) in contrast to SU86.86 cells in which IL-4 exposure resulted in a marked increase after 24 hours with an amplification of B7-H3 expression after 48 hours (Fig. 4D). Thus, in contrast to Panc-1 cells, treatment with IFN-γ and more pronounced treatment with IL-4 increased expression of B7-H3 on the surface of SU86.86 cells.

### B7-H3 protein expression correlates with the number of CD8+ T cells in human pancreatic cancer tissues

**Figure 5:**
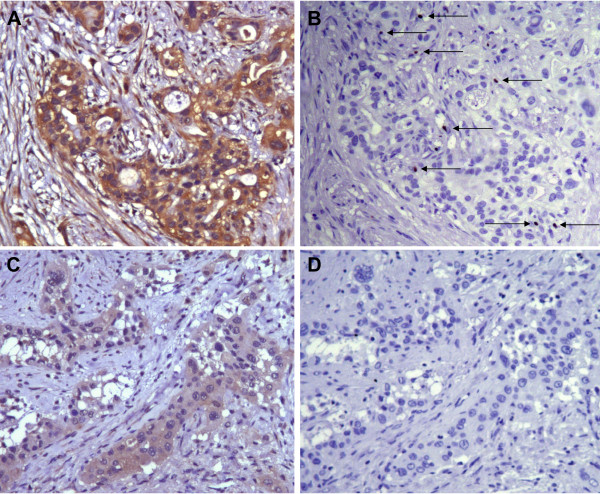
**Representative immunohistochemical stainings for B7-H3 (A, C) and CD8 (B, D) in pancreatic cancer tissues**. (A) Pancreatic cancer tissue section with strong B7-H3 immunoreactivity. (B) Consecutive section with immunostaining for CD8 shows the infiltration of numerous CD8+ T cells (arrows). (C) Pancreatic cancer tissue section with weak tumor B7-H3 immunoreactivity. (D) Consecutive section with immunostaining for CD8 shows no CD8+ tumor-infiltrating T cells.

**Figure 6:**
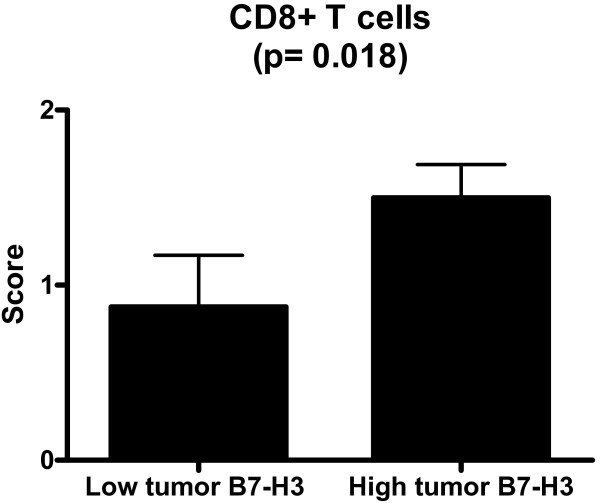
**Semi-quantitative analysis of CD8+ T cells in pancreatic cancer**. In areas with high tumor B7-H3 expression, the prevalence of CD8+ T cells was significantly increased compared to areas with low tumor B7-H3 expression (p = 0.018).

To determine the potential relevance of B7-H3 expression on the distribution of CD4+ or CD8+ T cells in pancreatic cancer, we first examined mRNA levels of CD4 and CD8 in 28 pancreatic cancer tissue specimens and 10 normal pancreatic tissue specimens. Gene expressions were markedly increased in pancreatic cancer tissues compared to normal pancreatic tissues (CD4: 150 transcripts ± 28.1 in pancreatic cancer vs. 52 transcripts ± 12.4 in normal pancreas; CD8: 171 transcripts ± 30.2 in pancreatic cancer vs. 39 transcripts ± 6.4 in normal pancreas). B7-H3 mRNA expression significantly correlated with CD4 (p < 0.0001, Spearman rho 0.76) and CD8 mRNA levels (p < 0.0097, Spearman rho 0.48). To determine whether the expression of B7-H3 by pancreatic cancer cells influences the distribution of tumor-infiltrating CD4+ or CD8+ T cells, we performed immunohistochemical stainings for B7-H3, CD4, and CD8 of 20 randomly chosen pancreatic cancer tissue sections. Immunohistochemistry analysis revealed a significant correlation between the level of tumor B7-H3 expression and the number of tumor-infiltrating CD8+ T cells (p = 0.018; Fig. 5 and 6). No correlation could be observed between the level of tumor B7-H3 expression and the number of tumor-infiltrating CD4+ T cells.

## Discussion

Pancreatic cancer is one of the most aggressive human malignancies and still represents a major therapeutic challenge. Despite the infiltration of large numbers of immune cells into pancreatic cancer, the immune system fails to prevent disease development and progression. Recently, dysregulation of immune responses by aberrant expression of negative costimulatory molecules by tumor cells has been suggested to play a potential role in the evasion of tumors from the immune system [4,5]. For example, tumor B7-H1 expression has been shown to significantly correlate with negative outcome in several human malignancies, including pancreatic cancer [11,13-16]. B7-H3 is another member of the B7 family of costimulatory molecules which serves as an accessory costimulatory regulator of T cell responses following initial T cell priming. The exact physiological function of B7-H3 and especially its role in the development and progression of human cancers are still elusive.

In this study, we demonstrated that the cultured pancreatic cancer cell lines Panc-1, MiaPaCa-2, and SU86.86 constitutively express B7-H3. Moreover, we demonstrated that B7-H3 gene expression is upregulated in pancreatic cancer tissues in comparison to normal pancreatic tissues. Immunohistochemical analysis revealed B7-H3 protein expression by pancreatic cancer cells. Tumor cell-associated B7-H3 expression significantly correlated with better postoperative survival. Moreover, we showed that B7-H3 gene expression in pancreatic cancer tissues significantly correlated with the levels of CD4 and CD8. Further immunohistochemical analyses on the distribution of CD4+ and CD8+ T cells revealed the infiltration of CD4+ and CD8+ T cells into areas of B7-H3 positive pancreatic cancer cells. More importantly, the number of tumor-infiltrating CD8+ T cells significantly correlated with the levels of B7-H3 on pancreatic cancer cells. Therefore, one could speculate that tumor-associated B7-H3 expression might act as a positive regulator of antitumor response in pancreatic cancer. B7-H3 might trigger the infiltration of CD8+ T cells into cancerous tissues and might also enhance tumor immunogenicity by stimulation of tumor-infiltrating CD8+ T cells. These findings are in accordance with previous reports on the function of B7-H3. Chapoval and colleagues reported that human B7-H3 costimulates proliferation of CD8+ T cells, enhances the induction of cytotoxic T cells, and selectively stimulates IFN-γ production by T cells in the presence of T cell receptor signaling in vitro [17]. Further data in support of a positive costimulatory role of B7-H3 in the regulation of immune response come from an experimental study showing that acute and chronic cardiac allograft rejection can be reduced in B7-H3 knock-out mice [26]. Recently, B7-H3 has also been implicated as a potential stimulator of antitumor immunity. In vivo, transfection of B7-H3 into P815 mouse tumors led to tumor regression and amplification of tumor-specific CD8+ CTL response in syngeneic mice suggesting enhancement of tumor immunogenicity by preferential stimulation of CD8+ T cell responses [19]. Further evidence in favor of a possible tumor-protective effect of B7-H3 expression in cancers comes from a clinical study investigating the expression of B7-H3 in human gastric carcinoma. The majority of tumors showed B7-H3 expression in tumor cells. Moreover, the presence of B7-H3 positive tumor cells was associated with improved patient survival [27]. Although these results clearly implicate a tumor-protective effect of B7-H3, the exact physiologic/pathologic role of B7-H3 remains ambiguous, because B7-H3 has also been shown to inhibit CD4+ and CD8+ T cell proliferation and IFN-γ production mediated by anti-CD3 in mice [28]. Recently, single or duplicate constructs of the immunoglobulin-V-like and immunoglobulin-C-like domains of B7-H3 have been shown to downregulate both T cell proliferation and cytokine production in response to CD3/CD28-mediated costimulatory activation [29]. Further evidence that B7-H3 may serve as an inhibitor of antitumor immunity includes several clinical studies. In prostate cancer, Zang et al. evaluated the expression of B7-H3 in over eight hundred prostate cancer patients and reported that 93% of the examined tumors displayed aberrant B7-H3 expression [23]. High tumor B7-H3 expression was associated with disease progression and spread as well as with poor patient survival [23]. Accordingly, tumor cell and tumor vasculature B7-H3 expression significantly correlated with an increased risk of death from clear cell renal cell carcinoma [30]. In these studies, however, the functional aspect of B7-H3 expression was not tested in detail. For example, correlation of B7-H3 expression by cancer cells with tumor-infiltrating immune cells was either not tested or not significant. The reason for the contrasting effects of B7-H3 in cancer might also be related to varying counter-receptors involved in different tumor entities. So far, one receptor of B7-H3 has been identified [31]. Hashiguchi and co-workers have recently reported that murine B7-H3 specifically binds to triggering receptor expressed on myeloid cells (TREM)-like transcript 2 (TLT-2, TREML2), which is expressed on both CD4+ and CD8+ T cells [31]. Moreover, the authors demonstrated that stimulation with B7-H3 transfectants preferentially upregulated the proliferation and IFN-γ production of CD8^+ ^T cells [31]. In humans, however, a receptor for B7-H3 has not been identified yet.

Another possible mechanism involved in antitumor immunity includes the induction of tumor-suppressive cytokines. Here, we found that B7-H3 was significantly associated with IFN-γ levels. IFN-γ is an important pro-inflammatory Th1 cytokine and has been implicated as a mediator of an extrinsic tumor-suppressor mechanism in immunocompetent hosts. IFN-γ is usually produced by activated cytotoxic T lymphocytes, macrophages, and natural killer (NK) cells [32]. In mice, treatment with neutralizing monoclonal antibodies to IFN-γ resulted in a faster growth of immunogenic sarcomas that were transplanted into mice [33]. Furthermore, endogenously produced IFN-γ has been shown to be protective against transplanted, chemically induced, and spontaneous tumors [34]. Therefore, B7-H3 might act as an immunostimulatory agent through the induction of IFN-γ.

Mechanisms regulating B7-H3 expression are still unclear. The local cytokine milieu in the tumor microenvironment represents a potential regulator of B7-H3. Indeed, IFN-γ has previously been shown to enhance surface expression of B7-H3 on bone marrow derived dendritic cells and on monocytes [18]. Therefore, we investigated the potential effects of the Th1 cytokine IFN-γ and Th2 cytokine IL-4 on the expression of B7-H3 on cultured pancreatic cancer cells. We found that both IFN-γ and IL-4 treatment resulted in a marked increase of B7-H3 on SU86.86 but not on Panc-1 pancreatic cancer cells. Furthermore, we found that B7-H3 positively correlated with IFN-γ levels in vivo. Taken together, these findings suggest a potential involvement of IFN-γ (and IL-4) in the regulation of B7-H3 expression in pancreatic cancer.

## Conclusion

We demonstrated for the first time that B7-H3 is abundantly expressed in pancreatic cancer and that tumor cell-associated B7-H3 expression significantly correlates with prolonged postoperative survival. Moreover, we showed that B7-H3 expression significantly correlated with the number of tumor-infiltrating CD8+ T cells. Although further functional studies will certainly be required to better understand the exact role of B7-H3 in pancreatic cancer, our data suggest that B7-H3 might be involved in the induction of a specific antitumor immune response and that treatment with B7-H3 (e.g. by gene transfer) might represent a promising approach to improve the outcome of pancreatic cancer patients.

## Abbreviations

CTLA-4: Cytotoxic T lymphocyte-associated antigen-4; IFN-γ: Interferon-gamma; IL-2: Interleukin-2; IL-4: Interleukin-4; qRT-PCR: Quantitative real-time polymerase chain reaction.

## Competing interests

The authors declare that they have no competing interests.

## Authors' contributions

ML and DMH conceived and designed the study, analyzed the data and drafted the manuscript. ML and NG supplied tissue specimens and collected clinical data. ML, DMH, and MO carried out immunohistochemical stainings. ML, DMH, and IE were responsible for the evaluation of immunohistochemistry data. DMH and ML performed the FACS analyses. NG carried out the qRT-PCRs. ML, JK, and HF provided expert guidance throughout the preparation of the manuscript, and reviewed the manuscript.

All authors read and approved the final manuscript.

## Pre-publication history

The pre-publication history for this paper can be accessed here:

http://www.biomedcentral.com/1471-2407/9/463/prepub
